# Interleukins and Interleukin Receptors Evolutionary History and Origin in Relation to CD4+ T Cell Evolution

**DOI:** 10.3390/genes12060813

**Published:** 2021-05-26

**Authors:** Norwin Kubick, Pavel Klimovich, Patrick Henckell Flournoy, Irmina Bieńkowska, Marzena Łazarczyk, Mariusz Sacharczuk, Suniti Bhaumik, Michel-Edwar Mickael, Rajatava Basu

**Affiliations:** 1 Institute of Biochemistry, Molecular Cell Biology, University Clinic Hamburg-Eppendorf, 0251 Hamburg, Germany; n.kubick@uke.de; 2 PM Research Center, 20 Kaggeholm, Ekerö, 178 54 Stockholm, Sweden; klimovichpavlusha@gmail.com (P.K.); patrickchenckell@gmail.com (P.H.F.); 3 Institute of Genetics and Animal Biotechnology of the Polish Academy of Sciences, ul. Postepu 36A, Jastrzebiec, 05-552 Magdalenka, Poland; i.bienkowska@igbzpan.pl (I.B.); m.lazarczyk@igbzpan.pl (M.Ł.); m.sacharczuk@igbzpan.pl (M.S.); 4 Bevill Biomedical Sciences Research Building, The University of Alabama at Birmingham, Birmingham, AL 35294-2170, USA; sunitibhaumik@uabmc.edu

**Keywords:** interleukins, CD4+ T cells, evolution, Th17

## Abstract

Understanding the evolution of interleukins and interleukin receptors is essential to control the function of CD4+ T cells in various pathologies. Numerous aspects of CD4+ T cells’ presence are controlled by interleukins including differentiation, proliferation, and plasticity. CD4+ T cells have emerged during the divergence of jawed vertebrates. However, little is known about the evolution of interleukins and their origin. We traced the evolution of interleukins and their receptors from Placozoa to primates. We performed phylogenetic analysis, ancestral reconstruction, HH search, and positive selection analysis. Our results indicated that various interleukins’ emergence predated CD4+ T cells divergence. IL14 was the most ancient interleukin with homologs in fungi. Invertebrates also expressed various interleukins such as IL41 and IL16. Several interleukin receptors also appeared before CD4+ T cells divergence. Interestingly IL17RA and IL17RD, which are known to play a fundamental role in Th17 CD4+ T cells first appeared in mollusks. Furthermore, our investigations showed that there is not any single gene family that could be the parent group of interleukins. We postulate that several groups have diverged from older existing cytokines such as IL4 from TGFβ, IL10 from IFN, and IL28 from BCAM. Interleukin receptors were less divergent than interleukins. We found that IL1R, IL7R might have diverged from a common invertebrate protein that contained TIR domains, conversely, IL2R, IL4R and IL6R might have emerged from a common invertebrate ancestor that possessed a fibronectin domain. IL8R seems to be a GPCR that belongs to the rhodopsin-like family and it has diverged from the Somatostatin group. Interestingly, several interleukins that are known to perform a critical function for CD4+ T cells such as IL6, IL17, and IL1B have gained new functions and evolved under positive selection. Overall evolution of interleukin receptors was not under significant positive selection. Interestingly, eight interleukin families appeared in lampreys, however, only two of them (IL17B, IL17E) evolved under positive selection. This observation indicates that although lampreys have a unique adaptive immune system that lacks CD4+ T cells, they could be utilizing interleukins in homologous mode to that of the vertebrates’ immune system. Overall our study highlights the evolutionary heterogeneity within the interleukins and their receptor superfamilies and thus does not support the theory that interleukins evolved solely in jawed vertebrates to support T cell function. Conversely, some of the members are likely to play conserved functions in the innate immune system.

## 1. Introduction

The innate immune system is more ancient than the adaptive immune system. Lower invertebrates are capable of defending themselves against pathogens using three main mechanisms: (i) pattern recognition receptors that allow nonspecific recognition of microbes, (ii) a phagocyte response that helps them eliminate foreign microbes, and (iii) cellular and biochemical factors such as the production of antimicrobial peptides that lyse cells, and ROS that target DNA [1]. The adaptive immune system first appeared in lampreys and hagfish [2]. Vertebrates’ adaptive immune system recognizes antigens with high specificity and applies appropriate responses against them. This sophisticated mechanism is based on two main types of cells that first appeared in jawed vertebrates (i.e., T and B cells) [3]. These two cell types are capable of specifically recognizing an immense number of antigens due to the variation in the T cell repertoire [4]. Antigen-presenting cells present to T cells their antigen through major histocompatibility complex I or II. Upon recognizing the antigen presented to them using their TCR, T cells become activated, and they differentiate toward an effector, memory, or regulatory (e.g., Tregs) type, based on the types of cytokines they produce [5,6,7]. Interestingly, TCR and BCR have been identified in jawed vertebrates but not in lampreys.

One of the main functions of interleukins is the regulation of CD4+ T cell function. Interleukins (ILs) form a family of cytokine proteins that play an important role in immunomodulation [8,9,10]. Interestingly, ILs can induce changes in nearby cells, and hence can function in paracrine form. Th17 cells produce IL29, which mediates the Antiviral Competence in Psoriasis [11]. ILs can also affect their own cell. IL10 produced by Th2 cells was demonstrated to regulate their survival and action [10,11,12,13]. IL17A was shown to reduce Th17 cell pathogenicity via a negative feedback loop driven by induction of IL24 [14]. It is not yet known if ILs can perform any endocrine function. On the level of their own cells as well as nearby cells, ILs can perform their immune modulation function by binding to their respective receptors. ILs can also modulate growth, differentiation, and activation during an immune response [15]. It has been shown that Th17 cells differentiate from naïve T cells under TGFβ, IL6, IL1B, IL23, IL12, while Th1 is differentiated under IL12, Th2 is differentiated from naïve T cells under IL4, and Tregs are differentiated from naïve T cells under TGFβ alone. Interestingly ILs could also support T cells’ plasticity from one phenotype to another. For example, Tregs could be converted into Th17 cells under IL6 while Th17 could be converted to Th1 under IL1B, IL6, and IL23. Moreover, ILs also represent the tools with which T cells exert their effect, for example, while Tregs produce IL10 which is anti-inhibitory, Th17 produces IL17A and IL17F, which were shown to have a pro-inflammatory effect [16].

The evolutionary history and origins of ILs are not yet fully understood. Firstly, since most ILs are primarily functioning in an autocrine or paracrine fashion on lymphocytes (e.g., CD4+ T cells) and since lymphocytes first appeared in jawed vertebrates, it has been suggested that ILs are a jawed vertebrates innovation [17]. However, it has been shown that interleukin 1β (IL1β), IL6, and IL8 perform functions related to the innate immune system. Furthermore, it was demonstrated that human recombinant IL1 was able to reduce the stress response of biogenic amines in mollusks [18]. This observation suggests that IL1 might have a conserved non-immunological function in invertebrates. The IL1 superfamily and its receptors has been investigated in vertebrates [19]. However, its evolution in invertebrates is still unknown. IL6, as well as IL6R, have been hypothesized to appear in invertebrates [20]. Domeless protein has been suggested to have a similarity with IL6R [21]. However, the existence of any evolutionary link between these two proteins has not been yet investigated. IL17 has been suggested to originate from nematodes [22]. However, its origin and evolution selection have not been investigated. In addition, IL17R family evolution has been studied in vertebrates and lampreys [23,24]. However, its evolutionary history in invertebrates has not been yet explored. Several families that belong to the interleukins IL2 superfamily have been identified in fish such as IL2, IL7, IL9, and IL1 [25]. However, a complete investigation of IL2 interleukins’ evolutionary history among other species is still missing. Furthermore, an evolutionary investigation covered IL2R superfamily receptors including IL2RA and IL15RA [26]. However, other receptor families that belong to this superfamily such as IL7RA, IL21R, and CRLF2A evolutionary history are still unknown. Moreover, the analysis of the evolution of IL4, IL10 and IL28 superfamilies’ as well as their receptors is not yet complete. Secondly, ILs have 40 members. They have been grouped into seven families following the classification in [9]. However, various members remain unclassified. Furthermore, the evolutionary relationships between IL members has been only analyzed in humans. Whether these superfamilies’ share a common origin is not yet known. A third aspect that has not been investigated is the evolution of the interleukins in lampreys. Lampreys possess two cell types that have been accredited for controlling the adaptive immune system known as VLRs. VLRs seem to perform an analogous function to T cells [8]. However, investigating interleukins and their receptors evolutionary history in lampreys is still unknown. Fourthly, the inter-evolutionary relationship between interleukins and their receptors is not yet complete. Whether interleukins coevolved with their interleukin, preceded them, or the receptors emerged first has not been investigated. Whether any sort of coevolution is linked to CD4+ T cell evolution or function is also unknown. Taken together, these observations suggest that a thorough investigation of the interleukins’ evolutionary history and their receptors is crucially needed.

The main aim of this research was to investigate the evolution of interleukins and their receptors in relation to CD4+ T cell evolution. We built multiple sequence alignments and phylogenetic trees for each of these components. We traced the evolution of these cytokines in 12 different species from *Trichoplax adhaerens* to humans. Our investigation paints a complex picture of interleukins and their receptor evolution. We found that although interleukins are interlinked, they, as well as their receptors, are highly heterogeneous, without a single origin. Interestingly, several interleukin family ancestral sequences such as IL1 and IL6 are linked to TGFβ. TGFβ plays a fundamental role in CD4+ T cell differentiation. However, IL1 first appeared in mollusks long before CD4+ T cell divergence. The nearest common ancestor to IL17 seems to be SPZ, which is the ligand of the toll-like receptor in drosophila. IL1, IL6 and IL17 evolved under positive selection, suggesting they gained new functions during their long evolutionary history. This observation suggests that IL1, IL6 and IL17 original function could have been related to the innate immune system or non-immune system and they gained novel functions during the emergence of CD4+ T cells. We also found that lampreys possess IL17D and IL17E and as well as receptors IL17DR and IL17ER. This might indicate that IL17D and IL17E could have a comparable function to their vertebrates’ homologs. Interestingly, we found that the evolution of interleukins in relation to their receptors is heterogeneous. In higher vertebrates, there are various examples of coevolution of interleukins and their receptors, such as IL28 and IL2. However, we also identified two interesting phenomena. (i) In certain superfamilies, the receptors predate the emergence of their ligands, suggesting higher promiscuous behavior. (ii) In other cases, the ligands could predate the receptors. This might indicate that during evolution the receptors could shuffle their ligands. Taken together our results indicate that in certain instances, interleukins and their receptors emerged to facilitate CD4+ T cell function and in other instances, already existing receptors gained new functions relevant to CD4+ T cell purposes.

## 2. Methods

### 2.1. Database Search

The focus of this research was investigating the IL family’s evolution and their origins. Due to the diverse nature and long evolutionary history of the IL families, we used protein sequence alignment. Moreover, to make sure that our analysis is a reasonable representation of IL evolutionary history, we investigated the presence of each of the family members using rodents, Monotremes, Marsupialia, Diapsida, Actinopterygii, Salientia, Caudata, Arthropods, Nematoda, Lophotrochozoa, Tunicates, Cnidaria and Placozoa, which span more than 500 million years. Human IL protein families were used for BLASTP searches against the above-mentioned proteomes. The longest transcript was used in the analysis. Sequences were selected as candidate proteins if their E values were ≤1e−10. Sequences were further filtered by comparing the conserved domain in each family against the human ILs investigated.

### 2.2. Alignment and Phylogenetic Analysis

Phylogenetic investigation was done in two steps [2,27,28]. First, IL family amino acid sequences were aligned using MAFFT using the iterative refinement method (FFT-NS-i). After that, we employed, PHYML implemented in Seaview with five random starting trees to generate the final tree. The Neutrality test was performed using MEGA 6.

### 2.3. Ancestral Sequence Reconstruction (ASR)

We applied the maximum likelihood method to infer the ancestral sequence of each of the proteins investigated. For each protein, we used the ASR algorithm implemented in MEGA6 to build ancestral sequences [2,29]. This was followed by BlastP against the nearest earlier diverging organism. The BlastP outcome was only accepted if the E-value threshold was less than e−10. The evolutionary network for ancestral sequences was built using SplitsTree with a default setting and bootstrap value of 100 [30].

### 2.4. HHsearch

The HHsearch method was used to examine the evolutionary history of the ILs. Only proteins that have already diverged before the most ancient members of the family were considered as candidate parents [2,27].

### 2.5. Positive Selection

We used the maximum likelihood algorithm in PAML to identify ILs that have gone through positive selection [31]. In the first instance, respective complementary DNAs (cDNAs) were estimated using the backtranslation function on the EMBOSS server (https://www.ebi.ac.uk/Tools/st/emboss_backtranseq/ (accessed on 5 May 2021) [32]. Next, we employed the CODEML PAML v4.4 program to evaluate global and branch selection by calculating the substitution rate ratio (ω) given by the ratio of nonsynonymous (dN) to synonymous (dS) mutations [33].

## 3. Results

This study is concerned with estimating the evolutionary history of cytokines, in comparison with CD4+ T cell evolution. We used phylogenetic analysis of different orders of kingdom Animalia. Our results indicate that the IL families constitute a heterogonous group of proteins, with more than one origin. IL1β, IL2, IL4, and IL6 ancestral sequences have high similarity to that of TGFβ1, whereas the IL28 ancestral sequence is likely to have diverged from a common ancestor of BCAM. IL1 and IL6 have evolved under positive selection, suggesting that they gained specific properties in time with CD4+ T cells emergence. Whereas IL2, IL4, IL8, IL10, IL28, IL14, IL16, IL32, IL34, and IL40 diverged under negative purifying selection. Interestingly, two families diverged under positive selection in lampreys, namely IL17B and IL17E. Some of the families have an ancient, conserved function such as IL14 that first appeared in fungi, whereas others evolved rapidly and gained new functions, such as IL6. We also found that the receptors are less heterogeneous, where IL1R and IL17R seem to have diverged from a TIR containing protein ancestor during the divergence time of Mollusca. IL2R, IL4R, IL6R, and IL17R diverged from a fibronectin III containing protein. IL8R seems to have diverged from somatostatin. Overall, our results show that interleukins’ relationship with their receptors is complex, with IL2R coevolving with its ligand and emerging at the time of CD4+ T cells.

### 3.1. Workflow

**Figure 1:**
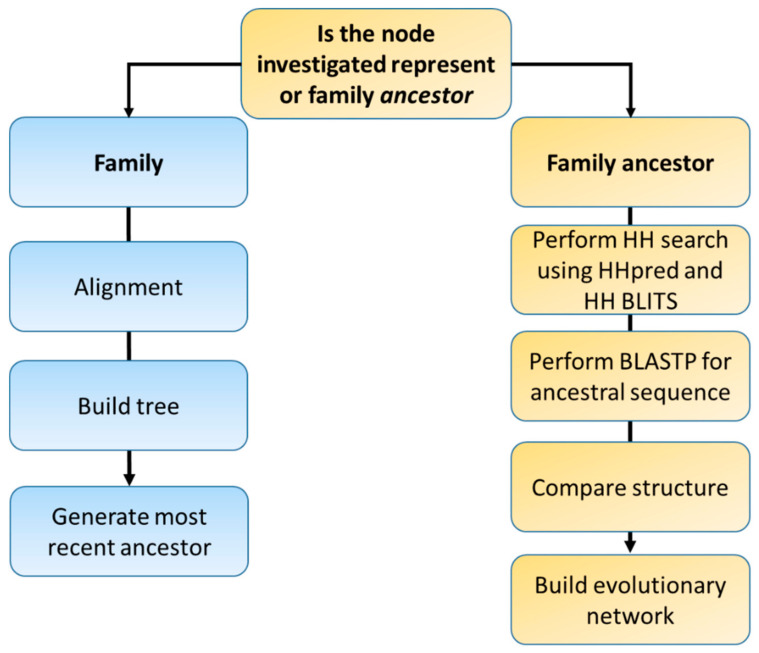
Workflow of the evolutionary analysis of the IL families. Each node was designated as a family or ancestral sequence node. The ancestral sequence for each family was used to generate an ancestral sequence for each superfamily. The ancestral sequence for the superfamily was utilized as an input for homology search. Finally, an evolutionary network was built.

We used the outline of the families presented in [9] to construct the phylogenetic analysis of ILs. To infer the relationship between various families, we categorized the family as an inner node. Outer nodes are the ancestral sequence of families. All ancestral sequences for each family were collected and homology investigation was done using Blast and HHsearch. The results from the homology investigation were used to construct an evolutionary network for each superfamily (Figure 1).

### 3.2. Evolutionary History of ILs Family

ILs have a heterogeneous evolutionary history. IL1 super family’s most ancient member is IL1β and it appeared in Lampreys, followed by IL18, which first appeared in fish. IL1F5, IL1F6, IL1F10, and IL38 appeared during the emergence of birds and reptiles, Il137 first appeared in Monotremata, while IL1A, IL33, and IL1F8 and IL1F9 appeared in Marsupialia. IL1β is not conserved in *Drosophila melanogaster*, *C. elegans* or *Nematostella*. IL2 super family has four ancient members that emerged during the Actinopterygii, namely IL2, IL7, IL15, and IL21. IL9 appeared during the emergence of amphibians, while IL2, IL7, and TSLP first appeared during the emergence of Monotremata. The IL4 super family consists of five members IL3, IL4, IL5, IL13, and CSF2. IL4 is the most ancient homolog of the IL4 superfamily as it first appeared during the emergence of the Actinopterygii, while IL3, IL5, and IL13 emerged during the divergence of Monotremata. CSF2 seems to be the most recent protein that belongs to this family as it emerged during the divergence of Marsupialia. We could not locate any IL6 superfamily members in invertebrates. The IL6 family consists of IL6, IL11, IL12A, IL 12B, IL23, IL27, IL30, IL31, IL35, CLCF1, CNTF, CSF3, CTF1, LIF, and OSM. IL6, IL11, IL12A, IL12B, IL23, and CNTF first appeared during the emergence of fish. CLCF1, CTF1, CSF3, and LIF first appeared in amphibians. IL27, IL30, IL35, and OSM first appeared in reptiles. IL31 first appeared in rodents. IL10 family consists of IL10, IL19, IL20, IL22, IL24, and Il26. IL10, IL20, IL22, and IL26 first diverged during the divergence of order Actinopterygii, while IL19 does not appear in birds, reptiles, or fish and it seems to have diverged during the emergence of order Monotremata. IL24 seems to be the most recent member of this family and it diverged during the divergence of Marsupialia. IL17 family is diverse, and it consists of the members IL17A, IL17B, IL17C, IL17D, IL25 (IL17E), and IL17F. We located IL17A, IL17B, IL17C, IL17D, and IL17F in mollusca. IL7E first appeared in lampreys. IL28 family consists of three members IL28a, IL28B, and IL29. Interestingly, whereas both IL28A and IL28B diverged during the emergence of reptiles. IL29 only appeared during the emergence of rodents. IL8, IL14, IL16, IL32, Il34, IL39, IL40, IL41, and IL42 are unclassified families. The most ancient of them is IL14, which is as ancient as Trichoplax and Fungi. IL32 and IL34 are the newest to diverge as they only appear in rodents.

### 3.3. Evolutionary History for ILs Receptors

**Figure 2:**
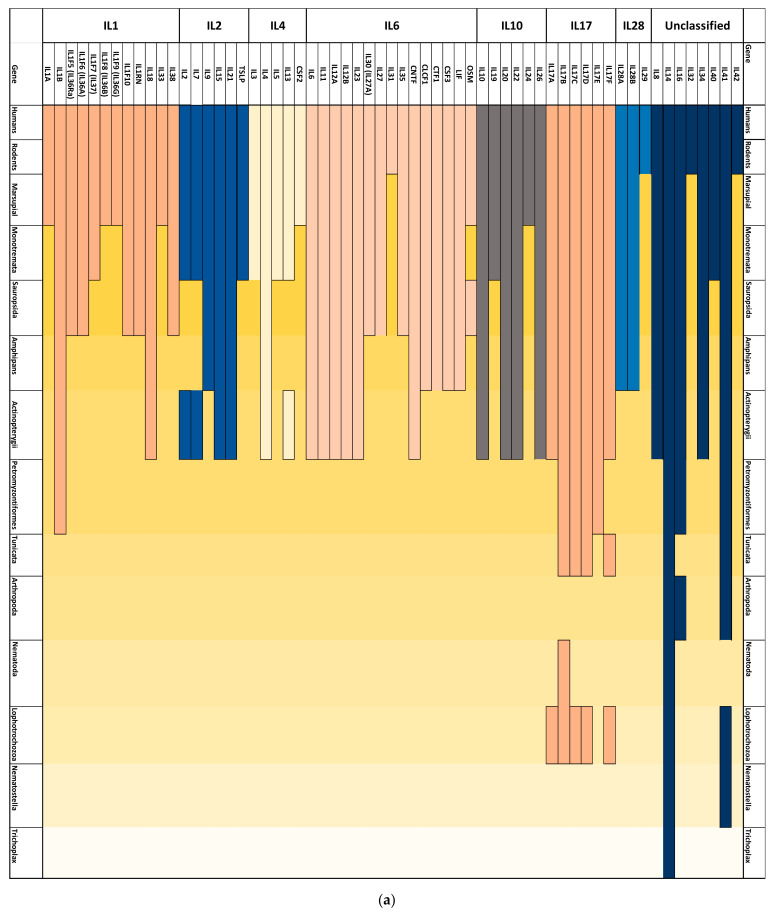

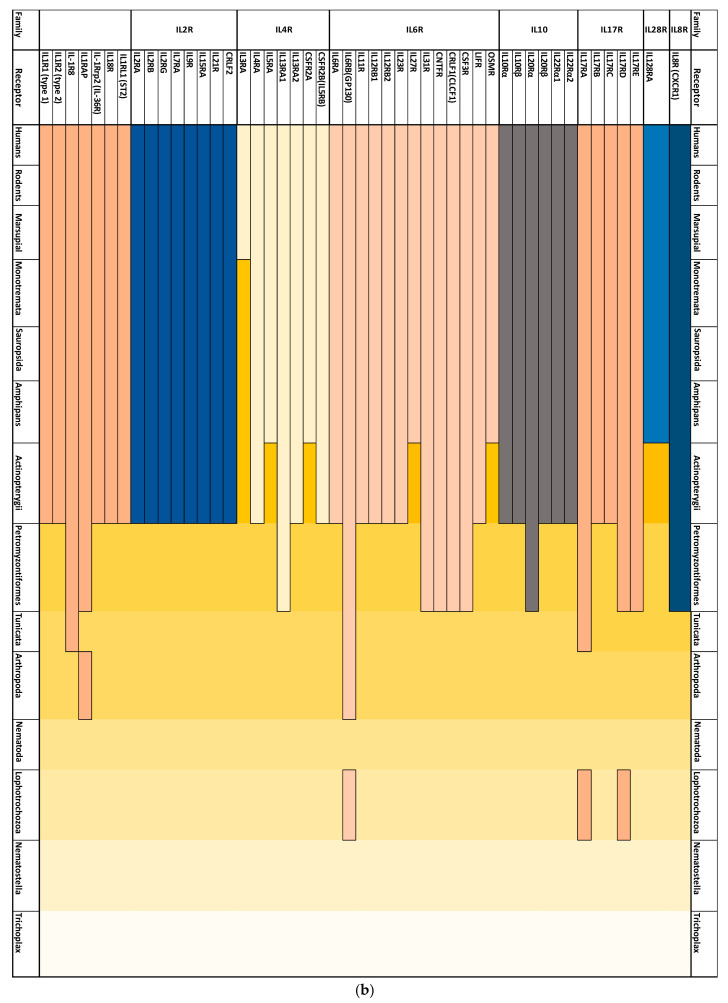
Evolutionary history of ILs and their receptors. (**a**) Interleukins evolutionary history; the most ancient member of ILs is IL14, which first appeared in Trichoplax and has an orthologs in Fungi. IL1B and IL17A, which both play critical roles in T cell function, first appeared in lampreys. Interestingly, IL6R appears in Drosophila. However, IL6 does not seem to have appeared in this species. (**b**) Interleukin receptors evolutionary history. IL17AR and IL17DR first diverged in Mollusca. IL-1R8 and IL1RAP first appeared in lampreys, together with IL13RA, IL6RB(GP130), CNTFR, clcf1(CRFL1), IL20Rα IL17R, and IL8R. As lampreys possess VLAR, VLARB, and VLARC cells and not T cells, IL1B and IL6, as well as these receptors, could be performing distinctive roles in this species.

Interestingly, in various instances, IL receptors do not follow the same patterns as interleukins. IL1R super family is evolutionary diverse. There are two types of IL1R, namely IL1R1 (type 1) and IL1R2 (type 2) [34]. IL1RAP is a co-receptor of type I [35]. IL1R8 (single Ig IL1-related receptor) also interacts with IL1R1 [36]. IL1A, IL1B, and IL1RN bind to both IL1R1 and IL1R2 [37,38]. We identified IL1RAP and IL-1R8 in lampreys but neither IL1R1 or IL1R2 (Figure 2b). IL1RAP has a homolog in insects (XP_021924169.1) (Figure 2b), while *Ciona intenstinals* posesses a IL1R8 homolog (XP_002122649.1). It is interesting to note that the nearest protein identified by BLASTp to the IL1R superfamily in invertebrates was Toll-like receptors. Toll-like receptors have been reported to exist in *D. melanogaster*, *C. elegans and N. vectensis* [39,40,41]. Moreover, IL1F5 (IL36Ra), IL1F6 (IL36α), ILF8 (IL36β), IL1F9 (IL36γ) and IL1F10 (IL38) share the same receptor (e.g., IL1Rrp2 (interleukin 1 receptor-like 2), also known as IL36R [37]. IL1Rrp2 seems to have diverged in Actinopterygii (Figure 2b). IL18 binds to IL18R, while IL37 requires the receptors IL18Rα and IL1R8 (SIGIRR). IL18R seems to follow the same evolutionary history as the more ancient of two interleukins (e.g., IL18) and it first appeared in Actinopterygii (Figure 2b). IL1RL1 (ST2), which is the receptor for IL33, has also diverged in Actinopterygii (Figure 2b). IL2R superfamily seems to have diverged during jawed vertebrates divergence. IL2 binds IL2R, which has three forms; IL2Rα, (CD25), IL2Rβ (CD122), and IL2Rγ [42]. We have found the homologs of IL2R to humans in Actinopterygii (Figure 2b). It is important to note that IL2RA is rare among Actinopterygii species with only *Archocentrus centrarchus* (Flier cichlid) possessing it. IL7 binds to the IL7 receptor, a heterodimer consisting of Interleukin 7 receptor α and IL2RG [43]. IL9 binds to the IL9 receptor complex, which is also a heterodimer that consists of IL9R and IL2RG [44]. Both IL7R and IL9R seem to have emerged during the divergence of Actinopterygii (Figure 2b). IL15 binds IL15 receptor complex that consists of IL15RA, IL2RB, and IL2RG [45]. IL15RA first appeared in Actinopterygii (Figure 2a). IL21 binds the IL21 receptor complex that consists of IL21R and IL2RG [46]. IL21R first appeared in Actinopterygii, similar to IL2R, IL7R, IL9R, and IL15R. TSLP binds a receptor complex composed of the CRLF2 and IL7RA. CRFL2 was also identified in Actinopterygii (Figure 2b). For the IL4R superfamily, IL3 binds to IL3RA and IL3RB (IL5RB) (CSF2RB) [47]. The most ancient homolog of IL3RA receptors was detected in the common wombat (*Vombatus* *ursinus*). IL4 binds IL4RA [48]. IL4RA diverged in Actinopterygii (Figure 2b). The IL5R receptor is composed of IL5RA and IL5RB (CSF2RB). In addition to being shared as a receptor for IL3 and IL5, IL5RB is also shared by colony-stimulating factor 2 (CSF2/GM-CSF). IL13 binds to either IL13RA in conjunction with IL4R, or it can bind to IL13RA2 [49,50]. IL13RA1 seems more ancient than its homolog IL13RA2 as it first emerged in lampreys, while IL13RA2 first appeared in fish (Figure 2b). CSF2 (GM-CSF) can bind to two forms of its receptor; namely CSF2RA and CSF2RB (IL5RB). CSF2RB (IL5RB) is the oldest of the two forms as it first appeared in fish (Figure 2a). The first member of the IL6R superfamily is the IL6 receptor and it consists of an IL6 binding α chain and a signal transducer, gp130, which is shared among the receptors for the IL6 related cytokine subfamily [51]. The IL6R superfamily is highly heterogeneous. IL6Rb (go130) first appeared in mollusks, while IL6RA diverged in fish. IL11R the receptor of IL11 also first diverged in fish. Similarly, IL12RA and IL12RB, which form the components of the receptor IL12R first diverged in fish [52]. IL23R, which is fundamental for CD4+ Th17 function diverged in jawed vertebrates, suggesting conservation of Th17 function in zebrafish and humans. IL27RA together with gp130 form IL27R, which is the receptor for IL27 [53]. IL31R, the receptor of IL31, first appeared in lampreys. CNTFR binds to CNTF and it first appeared in lampreys (Figure 2b). Similarly, CRLF1 (CLCF1), which binds to CLCF1, also first appeared in lampreys [54]. CSF3R binds to granulocyte colony-stimulating factor (CSF3) and it first appeared in lampreys. LIFR is the receptor of (LIF) and it first appeared in fish. OSMR, the receptor of oncostatin M, first diverged in birds. In the IL10R superfamily, IL10Ra and IL10Rb, as well as IL22Ra1 and IL22Ra2, diverged during the emergence of Actinoptyregii. Notably, IL22RA1 form a dimer with IL-10Rβ2 to form IL22 receptor, whereas IL22RA2 inhibits IL22 [55]. IL26R is a heterodimer, and it consists of two chains; IL10R2, and IL20R1 [56]. While IL20Rb appeared in Actinopterygii, IL20Ra appeared first in lampreys. IL19 binds to IL20RA and IL20RB, while IL24 binds to IL22R1/IL20R2 and IL20R1/IL20R2 [57]. For the IL17R family, IL17RA first appeared in mollusks. IL17RB and IL17RC first appeared in fish. The ligand for IL17RD has not been identified. However, IL17RD is as ancient as Mollusca and IL17RE first appeared in lampreys. Interestingly, IL17F uses IL17RA and IL17RC as its receptor and it does not seem to have its own receptor. The IL28 family has only one receptor (i.e., IL28AR [58]). IL28RA diverged during the emergence of amphibians. For the unclassified interleukins, IL8 has been found to be able to bind to CXCR1 (IL8R), which first appeared in lampreys [59].

### 3.4. Interleukin Families Origin

**Figure 3:**
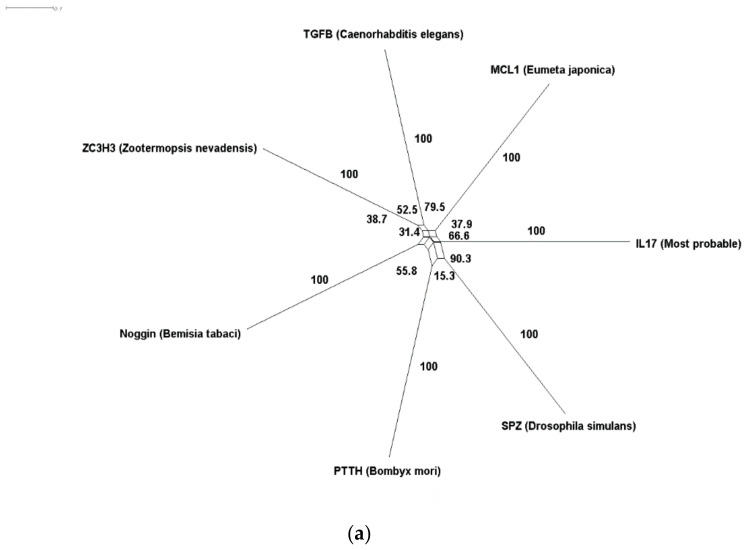

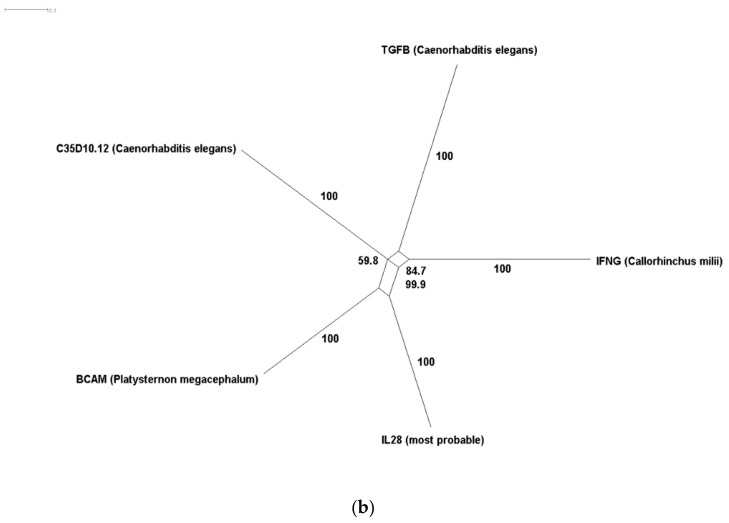
ILs and IL receptors do not share a sole origin. For each IL and IL receptor family, we identified the highest probable common ancestors using HHsearch and Blastp. Then we employed SplitsTree to estimate an evolutionary network with a bootstrap value of 100 using the identified sequences. (**a**) IL17 super family evolutionary network. Our workflow identified six different proteins as putative homologs to the ancestor sequence of IL17, namely, MCL1, SPZ, TGFβ, PTTH, Noggin, and ZC3H3. The nearest two homologs to IL17 are SPZ and MCL1. SPZ first appeared in *Drosophila*, while MCL1 appeared in Bagworm moth. (**b**) The nearest homologs of IL28 are IFNγ (Elephant shark), BCAM (Bighead turtle), C35D10.12 (Round worm) and TGFβ (Round worm).

Our results indicate that interleukins’ are a heterogonous family that does not share a recent single ancestor (Table 1). IL10 has three putative ancestors, namely MDM1, IFNγ, and TGFβ1. The IL4 family seems to share TGFβ1 with 99% probability according to the HHSearch algorithm. The same applies to the IL15 family (99.92%) (Figure 3). Interestingly, IL1, IL2, and IL6 seem to have diverged from a common ancestor of TGFβ, whereas the IL17 origin could be TGFβ1, SPZ, or MCL1. However, some families do not seem to be related to TGFβ such as IL28, which could be related to BCAM. IL14 also is not connected to the TGFβ line with high similarity to α-taxilin protein. The same applies to IL16, which seems to be connected to DLG4, IL32, which is more related to MMP25, and IL40, which is more related to synaptogyrin.

### 3.5. Interleukin Receptor Families Origin

Our results suggest that many of the interleukin receptor families diverged from fibronectin type-III domain-containing protein, such as IL2R, IL4R and IL6R. These results are supported by HHsearch (Table 1) as well as a comparison of structural components of the receptors with the putative protein candidates resulting from HHsearch. Investigations of conserved structural domains show a conserved fibronectin III domain in these respective receptors. IL17 and IL1 could be sharing a common ancestor with TIR. In agreement with our past reports, IL8R (CXCR1) seems to have diverged from the somatostatin group in rhodopsin-like receptors.

### 3.6. Neutrality Test and Positive Selection

We performed neutrality tests by computing the D value (Tajima’s Neutrality Test). We found that multiple families show positive values such as the IL10 family, IL1 family, IL17, IL8, IL32, IL34, IL40, and IL41. However, several families show negative values such as IL4, IL2, IL6, and IL14 (Table 2). We also performed positive analysis for lampreys Ils and ILs receptors (Table 3).

## 4. Discussion

Our analysis revealed a complex relationship between ILs’ evolutionary history and their function, which is not always related to CD4+ T cells. We investigated the relationship between various IL families and receptor families using phylogenetic, ancestral sequence reconstruction, homology search, and positive selection. Our findings can be summarized into three main points. First, ILs and IL receptors represent highly heterogonous and interlinked family groups. IL families have diverged at different points of emergence that at times predated CD4+ T cell emergence. For example, IL14 is as ancient as Trichoplax and fungi, whereas IL29, IL31, and IL32 only appear in rodents. Several family ancestor proteins are linked to TGFβ such as IL1, IL2, IL4, and IL6. However, due to the diverse nature of ILs, they are likely to have had multiple origins. On the other hand, IL receptors are less divergent. However several receptors emerged before CD4+ T cell emergence. IL17R and IL6R superfamilies, which are known to play critical roles in Th17 CD4+ T cells, first appeared in mollusks. IL1R seems to have first appeared in insects. IL4R and IL10R superfamilies first appeared in lampreys. IL28R first emerged in amphibians. IL1R and IL17R ancestors are linked to an ancestral TIR containing protein. IL2R, IL4R, and IL6R are linked to a fibronectin-containing proteins. IL8R diverged from the SOG group in rhodopsin-like family receptors. Secondly, there are various ancient ILs (e.g., IL14, IL16) that first emerged in invertebrates, which have evolved under negative selection (Table 2). These findings indicate that these IL families are likely to perform various functions that are non-specific for the vertebrate’s adaptive immune systems. Conversely, IL6 evolved under positive selection ω = 1.76 (*p*-value < 0.01) (Table 2). The IL6 superfamily is known to play an important function in Th17 induction in mammals. Our findings indicate that IL1 is not likely to be performing the exact function in lower vertebrates such as zebrafish. The third finding is that at least eight interleukin families and thirteen receptor families appeared in lampreys. Only two interleukin families exhibit positive selection, namely IL17B and IL17E (Table 3). The observation that certain families evolved under negative selection in lampreys indicates that these families might have a comparable function to those of the vertebrates (Table 3). Our results suggest that ILs’ evolutionary history–function relationship is a complex continuum with various families that started to appear in invertebrates.

ILs are a diverse family with more than one origin. For example, It has been suggested that IL28/29 cytokines represent a putative evolutionary link between type I IFNs and the IL10 family [60]. IL10 family evolution is recent, IL10, IL20, IL22, IL26 diverged in jawed vertebrates including fish (Figure 2a). CD4+ T cells are found in fish. It has been demonstrated that IL10 and IL22 perform analogous functions in fish to their vertebrates orthologs [61,62]. IL19 and IL24 are more novel, with the latter emerging during the divergence of Marsupialia. As IL19 is a homolog to IL120, it was difficult to decide which of the genes diverged first. However, based on our BLASTP analysis (Figure 2), we show that IL19 is more recent. IL19 is an immunosuppressive cytokine that plays an important role in supporting Th2 differentiation [63]. Since IL19 and IL20 have high similarity, and since IL19 can bind to the dimer receptor IL20R, it could be assumed that IL19 non-existence from fish is compensated with the existence of IL20 [64]. Interestingly, IL24 plays a major role in T cell proliferation [65]. Our results show that IL24 does not exist in lower vertebrates and thus seems to play a specific role in higher vertebrates including kangaroos, rodents, and humans. IL28 is a recent family; IL28A and IL28B have diverged during the emergence of amphibians, while IL29 only appears in rodents (Figure 2). IL29 plays an important role in supporting DCs to induce the proliferation of FoxP3-expressing regulatory T cells. Tregs are as ancient as jawed vertebrates [66]. Thus, the existence of IL29 only in rodents and humans could mean that it plays a specific role in higher vertebrates pertaining to Treg function that is unique to higher vertebrates. Interestingly, the IL28 family signals through a heterodimeric receptor consisting of IL28Rα (IFNλR1) which is responsible for ligand specificity, and IL10Rβ which is shared with all IL10 family members. The IL10R super family seems to have diverged from a common ancestor similar to the IFNα/β receptor (Figure 2b). IL28RA have two putative ancestral candidates namely fibronectin-containing III protein and IFNα/β that contains two fibronectin type II-like subdomains. Conversely, our analysis (Figure 3b) shows that the ancestral sequence of the IL28 family is more related to the BCAM gene than to IFNγ. Interestingly, the origin of various families has been linked to TGFβ1, namely IL1, IL2, IL4, IL6, and IL17. On the contrary IL14 (α-taxilin) is as ancient as fungi and has low similarity to the other ILs. Taken together, our investigation indicates that ILs are a heterogeneous family of signaling molecules that is not likely to have originated from a single precursor protein.

Our analysis demonstrates that various ILs perform multiple functions that are more ancient than the emergence of the adaptive immune system. We found that IL14, IL16, and IL41 and IL17 superfamilies have homologs in invertebrates (Figure 2a). ω values less than one for IL14 and IL16 indicate that these two cytokines could be performing a conserved function in both vertebrates and invertebrates (Table 2). Evidence supporting our findings of the conservation of IL16 function include IL16 ability to recruit and activate several cells other than CD4+ T cells such as monocytes, eosinophils, and dendritic cells. Meteorin-like IL, known as IL41, is a newly discovered cytokine that is highly expressed in macrophages. Interestingly, IL41 has been described as a hormone. However, whether it can function in an endocrine manner is still unknown. Furthermore, the evolution of IL6 and IL1 is an indicator of a low discriminatory activation of receptors in lower invertebrates. There have been various reports that demonstrated the existence of IL6-like protein and IL1-like protein in invertebrates. In particular, an IL6-like homolog was reported in *Drosophila* and is known as unpaired-3 (IL6-like) [67]. Unpaired-3 forms a signaling network with domeless (gp130-like), hopscotch (*Drosophila* homolog of mammalian Jak), and marelle (a *Drosophila* homolog of a STAT protein) to promote innate immunity. An IL6-like homolog was also reported in *Asterias forbesi* [68]. However, the similarity between humans’ IL6 or IL6 ancestral sequence with both *Drosophila* and *Asterias forbesi* is lower than the BLASTp threshold. We could not locate IL1B homologs in *C. elegans* (Figure 2). However, it was reported that *C. elegans* possesses a TIR-1 homolog that encodes Toll/IL1 resistance (TIR) domain protein. This TIR domain has been shown to play a role in resistance to microbial pathogens in *C. elegans.* In vertebrates, IL1β and TIR are known to play key roles in Th17 induction. IL1B does not exist in *Nematostella* or *Trichoplax*. However, IL1R was located in *Nematostella.* These results indicate that in lower invertebrates, receptors functioned in a promiscuous fashion [27,69]. As organisms evolved toward complexity, the uniqueness of activator ligands also increased.

Interestingly, our results show a complex evolutionary relationship between the interleukins and their respective receptors. The interaction between ligands and their receptors is the first step that connects CD4+ T cells to their environment. Three cases of ligand–receptor interactions have been reported, namely, coevolution, receptor predating ligand, and ligand predating receptor [70]. Our investigation identified these three categories. We found that IL2 and IL28 coevolved with their receptors. We also demonstrated that IL4R, IL6R, ILR8 and IL10R emerged before their ligands. Our results also identified cases where the IL family predates the receptor, such as the case of IL1, where the ligand–receptor interaction performs a fundamental role in CD4+ T cell differentiation and function. CD4+ T cells can change their phenotype and become activated or suppressed based on the environment. It could be noticed that the coevolved IL ligand and receptor both appear solely in vertebrates, which suggests possible conservation of function based on the maintained specificity of each ligand–receptor pair [71]. It is noticeable that in the case of IL4R, IL8R, and IL10R receptors, they first appear in lampreys, where receptive ligands appeared in fish (Figure 2). These observations could support the hypothesis that the emergence of these receptors in lampreys is related to the unique nature of the immune cells there. Alternatively, it could also be related to the low degree of specificity of receptors in lower vertebrates and in invertebrates [27,70].

Our results shed light on the unique immune system of lampreys. We have detected various cytokines and cytokine receptors in lampreys (Figure 2). Lampreys evolved with their specific phenotypes of immune cells such as VLRA (homologous α/β T cells), VLRB (homologous to B cells), and VLRC (homologous to γ/δ T cells) [72]. IL1B can perform diverse functions regulating Th17 differentiation by inducing alternative splicing of FoxP3 [73]. However, whether IL1B has an analogous role in lampreys is not yet known. Four homologous proteins of the IL17 family were found in lampreys (Figure 2). Interestingly, lamprey IL17D.1, which is homologous to mammalian IL17D.1, was found to be highly expressed in epithelial cells, VLRA and VLRC. IL17D.1 can bind to IL17RA.1 on the surface of VLRB. Treatment of lamprey blood cells with IL-17D.1 augmented transcription of various genes expressed by VLRB, suggesting a role of IL17D1 in VLRB activation. Our investigation demonstrated that IL17D was not under positive selection (Table 2). These findings advocate for a potential role of IL17 in regulating the function of the adaptive immune cells in lampreys, homologous to its vertebrate role. α-taxilin has been shown to bind to the syntaxin family and it plays a major role in vesicle trafficking. Our results indicated that IL14 was not under positive selection in lampreys (Table 2). This observation suggests that IL14 is playing a similar role in lampreys. IL16 is known to regulate T cell activation. Our selection analysis demonstrated that IL16 was not under positive selection. These results are in line with earlier reports that indicated a conserved role of IL6 in VLRC cells. However, whether lampreys possess any subpopulation in VLRC is not yet known. We have also located the immune-regulatory cytokine IL41 (known as meteorin-like protein) in lampreys. Our results show that it did not evolve under positive selection (Table 2). However, its function in lampreys is still unknown. Overall, out of the eight ILs present in lampreys, only two (IL17B, IL17E) evolved under positive selection. These results indicate that these lamprey conserved ILs are performing a function similar to their role in vertebrates.

## 5. Conclusions

In contrast to the classical belief that ILs are a jawed vertebrates’ innovation, our results indicate the ILs are a heterogeneous family of proteins that started in fungi (e.g., IL14) and continued to emerge during mammal emergence (e.g., IL29). Our analysis also indicates that neither ILs nor IL receptors seem to have diverged from a single ancestor. Interestingly, our results demonstrate that various ILs (IL1B and IL6) gained new functions that enabled them to influence T cell function. Overall, ILs are a diverse gene family that shares a common regulatory function of T cells. However, they have different evolutionary histories and origins.

## Figures and Tables

**Table 1:** Estimated origins of the IL families and their receptors.

Receptor Family	Origin
**IL1R**	TIR domain-containing protein (99.9%) Titin (99.89%)
**IL2R**	Fibronectin type-III domain-containing protein (99.69%) Granulocyte-macrophage colony-stimulating factor receptor subunit α (99.89%) cytokine receptor-like factor 2 isoform X1 (99.8%)
**IL4R**	Fibronectin domain-containing protein (99.9%) Insulin-like growth factor 1 receptor (99.89%) Interferon α/β receptor 1 (99.83%)
**IL6R**	granulocyte colony-stimulating factor receptor-like (99.99%) Leukemia inhibitory factor receptor α (99.99%) Fibronectin; FN3 DOMAIN, FIBRONECTIN (99.87%)
**IL10R**	Interferon γ receptor 1 (99.61%) Interferon α/β receptor 1 (99.63%) Granulocyte-macrophage colony-stimulating factor receptor subunit α; GM-CSF (99.65)
**IL17R**	Toll-like receptor 5; Toll-like receptor 5, (96.67%)
**IL28R**	Interferon α/β receptor 1 (Fragment) (98.7%) Fibronectin type-III domain-containing protein (98.44%)
**Unclassified** **(IL8)**	Somatostatin receptor (100%)
**Interleukins family**	**Origin**
**IL1**	Transforming growth factor β-1 proprotein (99.92%)
**IL2**	transforming growth factor β-1 proprotein (99.92%) Latency-associated peptide (99.91%) transforming growth factor β-3 (99.88%)
**IL4**	Transforming growth factor β-1 (99.68%) Growth/differentiation factor 8 (99.47%) Inhibin β A chain; Growth factor (99%)
**IL6**	transforming growth factor β-1 proprotein (99.92%)
**IL10**	Mouse double-minute 1 (99.71%) Interferon γ (96.73%) Transforming growth factor β-1-like (94.82%)
**IL17**	Transforming growth factor β-1 proprotein (99%) Spaetzle (98%) Zinc Finger CCCH-Type Containing 3 (97.1%) Prothoracicotropic hormone (96.5%) NOGGIN (95%)
**IL28**	Basal cell adhesion molecule (99.56%) Methyltransf_11 domain-containing protein (99.2%)
**Unclassified (IL8)**	IL1B (99%) IL36(99.7%) IL37(97.5%)
**Unclassified (IL14)**	α-taxilin (99%)
**Unclassified (IL16)**	Discs Large MAGUK Scaffold Protein 4 (99.56%) Amyloid β A4 precursor protein-binding family A member 2 (99.51%) Glutamate receptor-interacting protein 1(99.43%)
**Unclassified (IL32)**	MMP25 protein (99.5%)
**Unclassified (IL40)**	Synaptogyrin (99.28%) Platelet and Endothelial Cell Adhesion Molecule 1 (97.1%) Fragment Crystallizable RECEPTOR 1(97%)

**Table 2:** Neutrality test and positive selection test (ω) for ILs and ILs receptors (for neutrality test, D is significant if >2 or −2>).

Gene	Neutrality Test	ω	*p*-Value
IL1	0.99	1.83	<0.01
IL2	−2.4	0.73	>0.05
IL4	−1.4	0.83	>0.05
IL6	−0.8	1.76	< 0.01
IL10	2.4	0.46	>0.05
IL17	1.2	1.00	>0.05
IL28	n/c	0.9	>0.05
IL8	2.9	0.32	<0.01
IL14	−0.6	0.38	<0.01
IL16	0.4	0.48	<0.01
IL32	0.8	0.02	<0.01
IL34	1.5	0.43	<0.01
IL40	2.4	0.69	>0.05
IL41	1.5	0.31	>0.05
IL1R	4.0	0.61	<0.01
IL2R	1.6	0.49	<0.01
IL4R	1.07	0.3	<0.01
IL6R	2.02	0.38	<0.01
IL10R	2.01	0.84	<0.01
IL17R	1.5	0.39	<0.01
IL28R	3.5	0.69	<0.01
Unclassified (IL8R)	2.6	0.44	<0.01

**Table 3:** Positive selection test for ILs and ILs receptors expressed in lampreys.

Interlukin	ω	*p*-Value
IL1B	0.69	<0.01
IL17B	4.00	>0.05
IL17C	0.23	>0.05
IL17D	0.57	>0.05
IL17E	>10	<0.01
IL14	0.23	>0.05
IL16	0.40	>0.05
IL41	0.31	>0.05
Receptor	**ω**	***p*-value**
IL1R8	0.30608	>0.05
IL1RAP	0.46	>0.05
IL13RA1	0.1	>0.05
IL6RB	1.21	>0.05
IL31R	>10	>0.05
CNTFR	1.00	>0.05
CRFL1	>10	>0.05
CSFR	1.00	>0.05
IL20RA	0.1	>0.05
IL17RA	0.31	<0.01
IL17RD	1.00	>0.05
IL17RE	0.46	>0.05
IL8R	0.28	>0.05

## Data Availability

All data used is available as supplementary files.

## Supplementary Materials

The following are available online at https://www.mdpi.com/article/10.3390/genes12060813/s1.
