# Supplementary material for: Interleukins and Interleukin Receptors Evolutionary History and Origin in Relation to CD4+ T Cell Evolution
Source: Genes (Basel). 2021 May 26;12(6):813. doi: 10.3390/genes12060813 (PMC8226699; doi:10.3390/genes12060813)
Supplement: Supplementary file 1 [file genes-12-00813-s001.zip › Abbreviations table_adera.pdf]

Abbreviations table

Abbreviation	Description
GPCR	G protein-coupled receptor
IFN	Interferon
BCAM	Basal Cell Adhesion Molecule
TGFβ,	Transforming growth factor-beta
VLR	Variable Lymphocyte Receptor
